# Use of noncrystallographic symmetry for automated model building at medium to low resolution

**DOI:** 10.1107/S0907444911050712

**Published:** 2012-03-16

**Authors:** Tim Wiegels, Victor S. Lamzin

**Affiliations:** a European Molecular Biology Laboratory (EMBL) Hamburg Outstation, c/o DESY, Notkestrasse 85, 22603 Hamburg, Germany

**Keywords:** noncrystallographic symmetry, automated model building

## Abstract

A novel method is presented for the automatic detection of noncrystallographic symmetry (NCS) in macromolecular crystal structure determination which does not require the derivation of molecular masks or the segmentation of density. It was found that throughout structure determination the NCS-related parts may be differently pronounced in the electron density. This often results in the modelling of molecular fragments of variable length and accuracy, especially during automated model-building procedures. These fragments were used to identify NCS relations in order to aid automated model building and refinement. In a number of test cases higher completeness and greater accuracy of the obtained structures were achieved, specifically at a crystallo­graphic resolution of 2.3 Å or poorer. In the best case, the method allowed the building of up to 15% more residues automatically and a tripling of the average length of the built fragments.

## Abstract 2

Noncrystallographic symmetry is automatically detected and used to achieve higher completeness and greater accuracy of automatically built protein structures at resolutions of 2.3 Å or poorer.

## 1. Introduction

Macromolecular crystallography (MX) is the primary technique for the determination of structures of biomolecules at an atomic level of detail. MX has provided over 85% of the entries in the Protein Data Bank (PDB; Rose *et al.*, 2011 ▶; Berman *et al.*, 2000 ▶) and over 90% of those of complexes of proteins that are larger than 80 amino acids. The continuous exponential growth in the number of deposited PDB entries demonstrates the increasing demand for crystallographic three-dimensional structural information on biological macromolecules.

The current state of the methodological art is such that many challenging structure-determination projects come to a halt at a certain point. In particular, crystals of large proteins and their complexes may not diffract to a resolution at which an atomic model can be straightforwardly constructed. Indeed, even after semi-high-throughput sample screening, the crystals of currently studied projects diffract on average to about 4 Å resolution on synchrotron beamlines (Holton, 2005 ▶) and only a small fraction of the measured X-ray data results in a structure being deposited in the PDB. More precisely, the ratio between collected data sets and published structures is about 50:1 (Stroud *et al.*, 2009 ▶).

The apparent problem with low-resolution X-ray diffraction is that the amount of observed data that can be used for structure refinement and calculation of an electron-density map is limited. For example, for a protein crystal with 55% solvent content that diffracts to a resolution of 2 Å there are eight reflections per atom, whereas at a resolution of 4 Å this value decreases to one (Morris *et al.*, 2007 ▶). This lack of observations requires the use of additional restraints and causes smoothing of density maps and a loss of detectable atomic features. The development of automated structure-determination methods in MX has predominantly focused on high-resolution data, where bonded or at least angle-bonded atoms are resolved. Thus, the determination of low-resolution structures is usually beyond the normal operational range of crystallographic software and necessitates a large, if not excessive, amount of manual intervention.

Recent developments in the MX field do address automation in this resolution regime. Often impressive results are reported for low-resolution structure determination, although rarely can a complete structure be built without user intervention. For example, using the *PHENIX AutoBuild* wizard (Terwilliger *et al.*, 2008 ▶) it was shown that structures with data extending to resolutions around 2.8 Å could be built automatically to a completeness of higher than 80%. At a resolution of 3.3 Å the model completeness drops to 60%. A comparable performance is obtained for model building with *ARP*/*wARP* v.7.1 (Langer *et al.*, 2008 ▶; Morris *et al.*, 2003 ▶). Estimates from the *ARP*/*wARP* remote model-building web service suggest that structures at a resolution around 2.6 Å are typically built to a completeness of 80%. At 3 Å resolution the model completeness decreases to ∼75% and for cases with a resolution of 3.5 Å one may obtain a structure with only 65% model completeness. The *Buccaneer* software can build up to 80% of the model at resolutions down to 3.2 Å provided that the initial map correlation is higher than 0.6 (Cowtan, 2006 ▶).

All these and related approaches are limited by the quality of the initial phases; poorer phases generally result in less complete models. Reduction of the model completeness at medium-to-low resolution implies an increase in the number of shorter unconnected fragments built. There are different definitions of what is considered a correctly built model and discussion of these is beyond the scope of this paper.

Overall, automated interpretation of MX data in general and model building in particular in the resolution range 2.5–4 Å requires more research if it is to be generally successful. There is a need for novel approaches that will increase the completeness and quality of derived macromolecular structural information.

**Figure 1:**
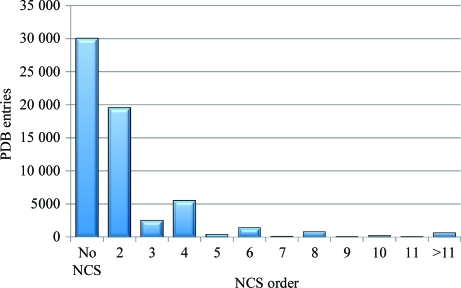
More than 50% of all structures in the Protein Data Bank (PDB) have NCS relations (derived from PDB data, March 2011).

More than 50% of the structures in the current release of the PDB (Fig. 1 ▶) contain a vast amount of intrinsic information: the so-called noncrystallographic symmetry (NCS). NCS occurs if there are multiple copies of subunits or their assemblies in the asymmetric unit of a crystal and these copies adopt almost the same fold and tertiary structure. The NCS order may be as high as 60 (*e.g.* lumazine synthase from *Salmonella typhimurium* LT2; Kumar *et al.*, 2011 ▶; PDB entry 3mk3); this results in 70% of all structural fragments in the PDB being involved in an NCS relation. Two types of NCS can be distinguished (Rossmann, 2001 ▶). An element which is independent in the sense of rotation is defined as ‘proper’. An example would be a molecule exhibiting an *N*-fold axis, with each element rotated by (360/*N*)° to the next one. ‘Improper’ NCS is referred to in the case of arbitrary rotation between two molecules in the same asymmetric unit.

The use of NCS has been an extremely valuable asset elsewhere in crystallographic structure determination (Rossmann, 1972 ▶; Bricogne, 1974 ▶; Kleywegt, 1996 ▶; Terwilliger, 2002 ▶). Perhaps its most frequent application is in density modification, in which NCS averaging helps to improve and extend phases to higher resolution as well as to reduce bias in cases where initial maps have been derived from an incomplete model (Kleywegt & Read, 1997 ▶). Here, the NCS relations can be specified by the user or derived from the determined heavy-atom positions. The electron-density map is segmented into areas related by NCS operators and for each operator a mask or envelope function is generated. Within these masks, the initial electron density for each operator is replaced by, for example, an average density over all NCS-related copies. A more recent use of NCS relations in MX involves their addition as restraints during structure refinement (Murshudov, 2011 ▶; Adams *et al.*, 2010 ▶); here, the stereochemical information from the regions of the protein chain that have been defined as NCS-related is added to the prior probability distribution in order to be used together with the observed structure factors in refinement.

**Figure 2:**
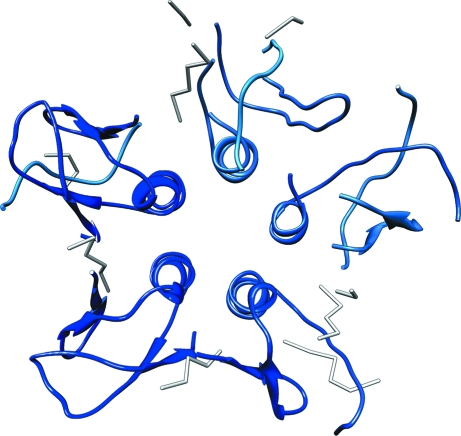
The result of standard automated protein model building of the test case shiga-like toxin (PDB entry 1c48) with *ARP*/*wARP* and X-ray data to 3.0 Å resolution. No NCS was used and each of the five subunits was built in a different way.

Here, we introduce a novel method for the automatic detection of NCS during automated model building with X-ray data at medium-to-low resolution. The derived NCS relations are in turn used to improve the model so that the built fragments can be extended and become more accurate. In essence, we exploit the fact that during automated model building NCS-related subunits are not built in exactly the same manner (Fig. 2 ▶). The causes of this can be manifold and include differences in local solvent accessibility or map/phase quality throughout the unit cell. During the process of model building each NCS-related copy of the structure thus contains information which may be lacking in another copy. Combining the information from several copies helps to increase the overall structural completeness. To circumvent computationally intensive examination of electron density, our method is based on the analysis of fragments of atomic models. In a number of selected examples, we demonstrate how the developed methodology is implemented in the *ARP*/*wARP* package as a dedicated and efficient *PNS Extender* (*Protein NCS-based Structure Extender*) module.

## 2. Methods

### 2.1. Data

In order to carry out computational tests, we used high-resolution structures from the PDB as well as a number of structures that had been submitted to the *ARP*/*wARP* model-building web service and made available for testing purposes. A good representative example is the 1.6 Å resolution structure of the B subunit of a mutated shiga-like toxin (PDB entry 1c48; Ling *et al.*, 1998 ▶). The molecule is arranged as a homopentamer, with each subunit composed of 69 residues. This structure was predominantly used for the basic development of the method. The full test set used for subsequent examination of the effectiveness of the method consisted of 11 multimeric structures that were determined at resolutions ranging from 2.4 to 3.2 Å with asymmetric unit contents of between 300 and 2300 residues in 2–10 NCS-related subunits. The structures were characterized by varying secondary-structural content, so that there were predominantly helical, stranded and mixed α–β models.

### 2.2. Clustering of transformations between chain fragments and identification of NCS-related copies

The method is based on the comparison of partially built protein-chain fragments of an intermediate model during the *ARP*/*wARP* model-building protocol (Langer *et al.*, 2008 ▶). With *ARP*/*wARP*, an initial protein model, a set of ‘free atoms’ with no chemical identity or a mixture of the two (the hybrid model) undergoes iterative transformation. In each building cycle some ‘free atoms’ gain chemical identity and are recognized as part of a protein-chain fragment, while others remain free. The evolving hybrid model combines two sources of information: it incorporates chemical knowledge from the partially built model and the free atoms continue to interpret the electron density in areas where no model is yet available.

**Figure 3:**
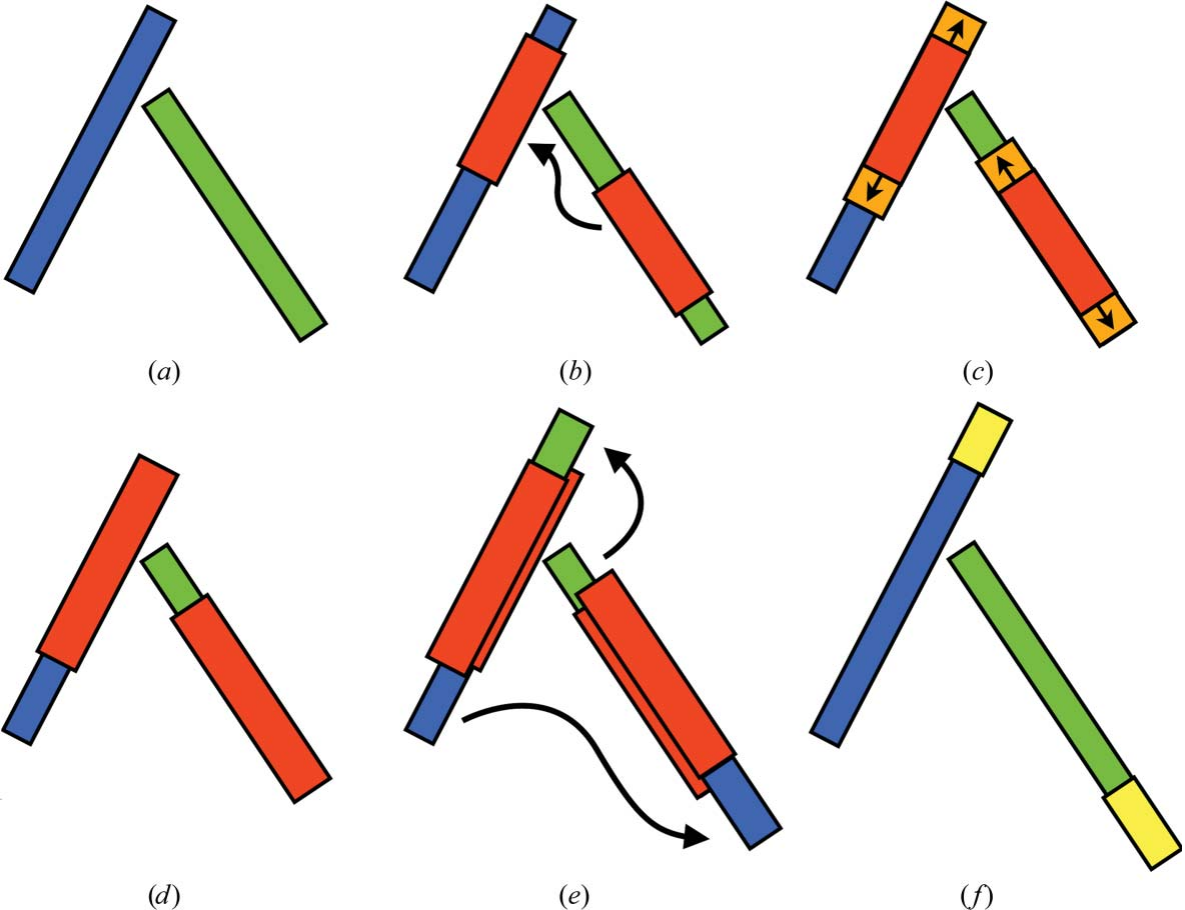
Workflow of the *Protein NCS-based Structure Extender* (*PNS Extender*). Intermediate partial models are examined for symmetric dependencies between stretches of two fragments (*a*). An initial match is found between green and blue regions (*b*, red blocks). The initial match is extended in both directions of the chain fragments (*c*, orange blocks). Once the extension is finished and the r.m.s.d. between the extended matches (red blocks in *d*) is still below the acceptance threshold, each extension (*e*, overlayed blocks) is NCS-transformed, as shown by arrows, onto the other fragment. Finally, longer extended green and blue fragments are obtained (*f*) and their extended parts (*f*, yellow blocks) are input as guides for protein-chain tracing.

The first step of the *PNS Extender* module (Figs. 3 ▶ *a* and 3 ▶ *b*) involves an analysis of the partially built protein-chain fragments for possible symmetry-related dependencies. Each stretch of a fixed number of C^α^ atoms of each chain fragment is least-squares superposed with each stretch of the same length of every other fragment.

Pairs of stretches which superimpose with an r.m.s.d. between C^α^ atoms below a fixed threshold (0.4 Å for resolutions higher than 2.8 Å, otherwise 0.5 Å) are selected for further analysis of their rotational components. We use quaternions to compare the rotations of matched fragments following the formulation of Kearsley (1989 ▶). Quaternions can be seen as vectors in four dimensions extending from the origin onto the surface of a three-dimensional hyper-sphere with unit radius. Thus, a quaternion has unit length and only three parameters; the fourth one can be computed from the other three. These and other properties (Mackay, 1984 ▶) make quaternions a convenient tool for the description of three-dimensional rotations as well as for animation in computer graphics, computer vision, robotics *etc*. The difference between two rotations can be determined by calculating the dot product between the two respective quaternions. If the difference is below 5°, the two rotations relating respective pairs of C^α^-atom stretches are deemed to belong to the same cluster. A rotation difference of 5° was chosen to allow some variation in the derived NCS operators. This parameter is dependent on the accuracy of the built fragments and may vary as a function of resolution. Highly populated clusters of rotations point to a correspondence between NCS-related copies. Since only pairwise NCS relations are considered, the method is able to detect both proper and improper symmetries.

### 2.3. Improving structural information by transformation of NCS copies

To find the longest continuous region of the NCS match between two fragments, we adjust each initial overlapping stretch (as shown in Fig. 3 ▶ *b*) by extending the matching region in both directions along the chain (Fig. 3 ▶ *c*). During the extension we recompute the r.m.s.d. over the increased length, *L*_ext_. Should the r.m.s.d. exceed a predefined threshold of 0.2*L*_ext_ Å, the inspected NCS match is not considered further. This helps to reduce false positives by avoiding arbitrary or unlikely matches. Once extension is complete, the remaining ‘tails’ (Fig. 3 ▶ *d*, blue and green ‘leftover’ tubes) are considered on both sides of the overlap region. All C^α^ atoms from the tails of each fragment are NCS-transformed to the end part of the corresponding fragment (Fig. 3 ▶ *e*). Should there be stereochemical clashes (defined as two atoms being at a distance of less than 0.7 Å from each other) between an NCS-transformed atom and any other atom from existing protein-chain fragments, the former is deleted.

### 2.4. Weighting the found fragments

The extensions obtained from the tails of partially built protein-chain fragments (Fig. 3 ▶ *f*) are not error-free and therefore need to be weighted according to their estimated accuracy. The errors may originate from the detection of matches between common structural motifs, such as helices, which may not necessarily be related by NCS. In addition, in the case of an NCS order higher than two, more than one copy of the same extension can be obtained (*e.g.* for a trimer fragment 1 transferred to fragment 2 and also fragment 3 to fragment 2). Therefore, we implemented a weighting scheme for extended fragments (Fig. 3 ▶ *d*). This weighting accounts for the clustering of initial rotational transformations (§[Sec sec2.2]2.2 and Fig. 3 ▶ *b*), as well as the preliminary fragment extension (§[Sec sec2.3]2.3; Fig. 3 ▶ *c*), 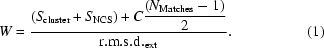
This equation contains two parameters reflecting the relative size of the cluster of rotations: the cluster size compared with all other clusters (*S*_cluster_) and the cluster size compared with the expected size of an NCS-related part of the molecule (*S*_NCS_). These parameters can take values between 0 and 3 as follows. *S*_cluster_ is 0 if the considered cluster is smaller than the average cluster size, 1 if it is larger, 2 if it is twice as large and 3 if it is three (or more) times larger. Similarly, *S*_NCS_ is 3 if the considered cluster is larger than the expected size of an NCS-related part of the structure, 2 if it is half the size, 1 when it is a quarter of the size and otherwise 0. *N*_Matches_ amounts to the total number of initially superimposed stretches (Fig. 3 ▶ *b*) that have led to the construction of the extended fragment (Fig. 3 ▶ *c*). *C* is a scaling coefficient that is usually set to 1. The denominator r.m.s.d._ext_ can take values between 0 and 0.2*L*_ext_ as described in §[Sec sec2.3]2.3.

Typically, the weights vary between 0 and 100. The higher the weight, the more likely it is that the extension fragment is a valid NCS hit. The weights are then used in further steps of the procedure to rank the extension fragments. A limited number of top-ranked extensions are fed back into the further model-building process.

### 2.5. Use of identified NCS-based fragment extensions for model building

**Figure 4:**
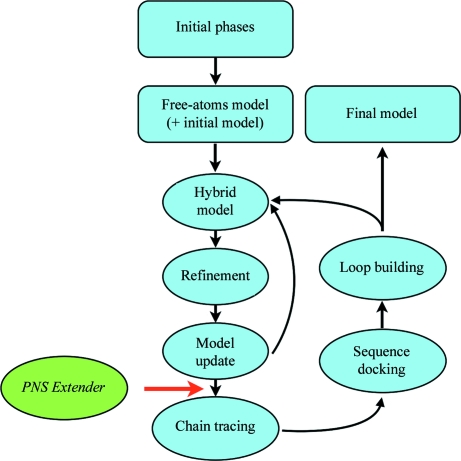
Flowchart of *ARP*/*wARP* protein model building, including automatic NCS detection and its use for extension of the model.

Within the *ARP*/*wARP* workflow, the NCS-extension method is applied to the intermediate model as depicted in Fig. 4 ▶. Specifically, the obtained NCS-based extensions of protein-chain fragments are added to the current hybrid model before the main-chain building block. In the current v.7.2 of the *ARP*/*wARP* software suite, the method is invoked by default if the resolution of the data is poorer than 2.3 Å.

### 2.6. Derivation of NCS-based stereochemical restraints

The information about the identified NCS-related copies is also used to construct stereochemical restraints for the iterative refinement of the model with *REFMAC* (Murshudov *et al.*, 2011 ▶). This step ensures that the parameters of the built hybrid model are adjusted to better fit the experimental data and *a priori* stereochemical expectations. The application of NCS restraints in *REFMAC* is realised by specifying chains or fragments of chains that are related through NCS operations. To ensure that we only formulate NCS-based restraints for highly reliable chain fragments, we do so only for extended overlaps that are more than 15 residues long. Furthermore, since we generate NCS instructions for both main-chain atoms (medium restraints) and side-chain atoms (loose restraints), we only apply these to fragments that *ARP*/*wARP* has docked into the sequence. By default, such NCS restraints are generated if the resolution is poorer than 2.3 Å.

### 2.7. Designed tests

We initially tested the ability of the *PNS Extender* module to automatically identify and apply NCS relations to the appropriate parts of the model: the ‘exclusion’ test. A single model, the mutated shiga-like toxin B subunit (PDB entry 1c48), was used for this purpose. We artificially fragmented the structure by cutting out parts of the model in order to mimic real cases, in which intermediate models may contain a large number of unconnected fragments. To generate cases with various degrees of fragmentation, we built ten differently fragmented structures. Starting from the complete structure, we successively deleted 5% of residues from each model, with 95% of the structure left in the first test case, 90% in the second case through to 50% in the tenth case. The models were fragmented by cutting out blocks of residues (15–30 amino acids; see Fig. 6*b* for the seventh case with 65% of the model left) from different parts of the structure.

Subsequently, we evaluated the extent of the improvement observed when all of the test structures described in §[Sec sec2.1]2.1 were built using the automated model-building protocol of *ARP*/*wARP* including the *PNS Extender* module. Each protocol was executed with five cycles of model update and refinement after each of the ten model-building cycles. For these tests, we used *ARP*/*wARP* v.7.2, *REFMAC* v.5.5.0109 and *CCP*4 v.6.1.13 (Winn *et al.*, 2011 ▶).

## 3. Results

### 3.1. The importance of scoring the fragment extensions

**Figure 5:**
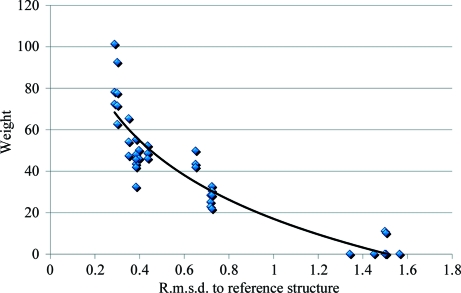
Estimated reliability of the derived weights used for NCS extension and the accuracy of the obtained extended parts of the model.

To prove the validity of the weighting scheme (as described in §[Sec sec2.4]2.4), r.m.s.d. values between the NCS-extended parts of a model (extension fragments) and a reference structure were calculated for a spectrum of cases. These were compared with the weights assigned to the extensions (1). As expected, small deviations in the reference structure (of 0.2 Å or less) corresponded to extensions with high weights (Fig. 5 ▶). Extensions with low weights resulted in larger deviations (of ∼0.7 Å or more) from the reference structure. This indicates that extensions with higher weights are indeed more accurate.

### 3.2. Effect of the completeness of the structure in the absence of the coordinate error

**Figure 6:**
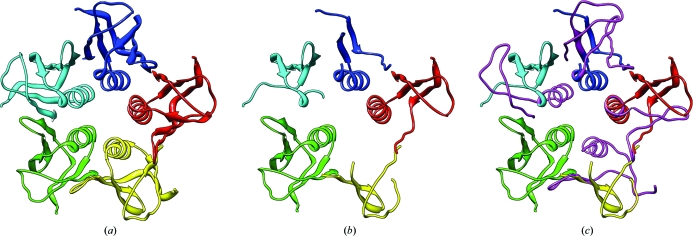
Validation of method properties: exclusion test. (*a*) The original structure (PDB entry 1c48); (*b*) the same structure with 35% of all residues excluded. In (*c*) all missing residues in (*b*) were retrieved using *PNS Extender*.

For each of the artificially fragmented test structures from the mutated shiga-like toxin B subunit 1c48 (Figs. 6 ▶ *a* and 6 ▶ *b*) we used *PNS Extender* to retrieve the missing C^α^ atoms. Each structure was checked against the full reference model to examine the accuracy of the retrieval. For the first seven test structures (5–35% of the residues excluded) our method rebuilt the complete model with an r.m.s.d. to the C^α^ atoms of the reference structure of 0.33 Å or better (Figs. 6 ▶ *b* and 6 ▶ *c*). As would be expected, the accuracy of the retrieved parts of the structure decreased gradually as a larger fraction of the model was excluded (Table 1 ▶). For the last three cases it was not possible to retrieve the complete structure, although the accuracy of the retrieved parts was still very high.

### 3.3. Incorporation of *PNS Extender* into *ARP*/*wARP* protein model building

The main application of our NCS-based structure-extension method is for improving model completeness/fragmentation at medium-to-low resolution, specifically in the *ARP*/*wARP* protein model-building protocol. The CPU requirement on a modern desktop computer is dependent on the size of the structure, its NCS order and the degree of fragmentation of the starting model. The current software implementation is reasonably fast – it takes less than a second for a moderately fragmented homodimeric structure (2 × 331 residues) – which is only a small additional overhead compared with model building without NCS. The generation of NCS restraints for refinement is even more rapid, since only the longest overlaps between NCS-related fragments need to be identified.

For evaluation of the method, we tested a wide range of parameters, including the r.m.s.d. threshold below which pairs were deemed to match, the initial length for the identification of NCS-related fragments and the number of located fragments to be fed back into the modelling process (ranked according to the weights described in §§[Sec sec2.4]2.4 and [Sec sec3.1]3.1), as well as a number of different protocols, including one with an option to remove short matches that have been identified as helix-only (using the algorithm of Zhang & Skolnick, 2005 ▶). We further tested NCS extension with and without the use of the best-ranked NCS relations as restraints in *REFMAC*.

**Figure 7:**
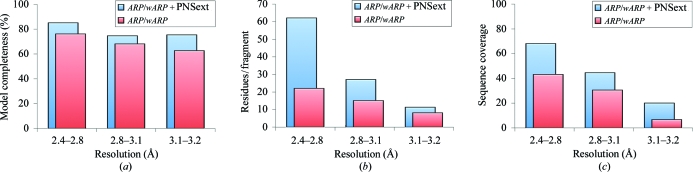
The best results for tests with variable r.m.s.d. thresholds for acceptance of identified NCS matches (0.4/0.5 Å) and a variable amount of top-ranking fragments to be fed back into the model-building process. (*a*) Average completeness of the built model. (*b*) Average length of built fragments. (*c*) Residues that have been assigned to sequence.

In all cases we observed a larger number of residues built and a greater average length of protein-chain fragments; the relative improvement in model building was almost independent of the resolution of the data within the inspected range. Notably, the resulting models became less fragmented, which should simplify their completion by manual intervention. We further noticed improved sequence coverage (the number of built residues that have automatically been docked to the sequence) in all cases. There were also decreases in *R* factor of up to 7.5%, increases of up to 15% in model completeness at a resolution around 3.2 Å and a tripling of the average length of the resulting protein-chain fragments (Fig. 7 ▶). The average length of the built fragments was more than doubled for the test cases with resolutions from 2.4 to 2.8 Å.

Subsequently, we identified the parameters of the protocols that gave the best improvement for all tested structures at various sizes and data resolutions. We observed that during protein-chain tracing smaller fragments are more likely to contain mistakes. This could be attributed to the fact that the connectivity and nonbranching nature of the protein chain serves as an extremely powerful constraint in model building with *ARP*/*wARP* and helps to eliminate incorrect chain diversions. Use of small chain fragments introduces noise in the derivation of the NCS operators and smears out their clusters during the identification of NCS-related copies. This in turn disturbs the ranking of the NCS matches and ultimately may result in incorrect extensions being sent back to the model-building process and thus the introduction of additional complexity in the chain-tracing procedure. In order to avoid such problems, we set the minimum number of residues of C^α^ stretches used for initial least-squares superposition to the current average length of built chain fragments in the structure.

We also found that a lower r.m.s.d. threshold for acceptance of NCS matches provided better results at medium than at lower resolutions, as the accuracy of the matches is likely to correlate with the coordinate error. Thus, for data with resolution better than 2.8 Å the threshold was set to 0.4 Å and for poorer resolution data it was set to 0.5 Å. More elaborate dependencies may be sought in the future. Additionally, only a limited number of top-ranked extensions (typically three) are fed back into the model-building process.

Overall, use of the method with the optimized parameters resulted in models with 5% higher model completeness, 25% longer chain fragments and 10% greater sequence coverage than models built with the *PNS Extender* module switched off.

## 4. Conclusions

The obtained results prove the general benefit of NCS-based extension of the protein model during building and refinement. A protocol has been developed that provides notable improvements within the resolution range 2.4–3.2 Å. Especially at resolutions around 3.1–3.2 Å, the use of the method gave rise to a 20% increase in the chain length of the fragments; the fragment length was generally greater than ten residues, which is often seen as an indicator of a ‘good’ model. Further optimization of the protocol parameters will certainly provide further enhancement. Since *PNS Extender* is invoked within *ARP*/*wARP* web-based model building (as of v.7.2), there should be ample opportunity for its continuous evaluation using a wide variety of cases where data are available.

In the ‘exclusion’ test scenario the chain fragments are free of phase-dependent coordinate error. Also, there are no mistakes in the traced chain fragments such as route shortcuts or spurious loops, meaning that the test case was somewhat idealized. There may, however, be inherent differences between NCS-related parts of the structure, as there are between chain *E* and all other chains in the model of mutated shiga-like toxin B subunit. Indeed, the NCS operators are rarely exact across all copies of a fragment and the reader is referred to Tête-Favier *et al.* (1993 ▶) or Poon *et al.* (2010 ▶) for a discussion on this topic. Nevertheless, in the exclusion test the retrieval of the full pentameric structure to a very high accuracy (Table 1 ▶) was possible even when the initial model was highly fragmented and contained only 65% of its C^α^ atoms. We thus estimate that in the best-case scenario, in which all NCS matches are accurate and there are no coordinate errors, it may become possible to retrieve the full structure of a protein at 3.5 Å resolution in a single building cycle, even with the current performance of the *ARP*/*wARP* protein model-building module.

The NCS-extension method and the way in which its results are used in the protein model-building protocol has certain advantages over other possible approaches. For example, plain averaging of the coordinates of the NCS-related copies may not be the best option as it introduces a certain degree of model bias and may also move some parts of the averaged model out of the density. In our implementation, the NCS-extended fragments are only used as potential C^α^ seeds (suggestions) to *ARP*/*wARP* for building longer chain fragments. Therefore, the method is not expected to build parts of the structure that lack support for coordinate placement in terms of electron density and plausible stereochemistry. Thus, if some parts of the NCS-related copies of the model are similar then their convergence to similar conformations will be accelerated by the method. If, in contrast, there are genuine differences between the NCS-related copies then the NCS-extended suggestions will not match the density and will likely not be used to build the chain.

One main conclusion is that the accuracy of NCS extension depends predominantly on the completeness of the initial model, its degree of fragmentation and the coordinate accuracy (*i.e.* very good results will still be obtained for many residues missing from a nearly correct model). Another conclusion is that the use of NCS extension in model building (at least in the current implementation of *ARP*/*wARP* protein chain tracing) is always advantageous, but the degree of improvement depends even more strongly on the completeness, fragmentation and correctness of the model, which all in turn depend on the quality of the phases and the data.

Although the method has been developed for proteins, the symmetric nature of complementary strands in DNA calls for an investigation of its applicability to model building of polynucleotide structures.

## 5. Software availability

The NCS-based method for automatic detection of NCS operators that are used for the extension and connection of partially built protein-chain fragments as well as for the generation of NCS restraints for *REFMAC* refinement has been incorporated into the *ARP*/*wARP* software project (v.7.2), which is available at http://www.arp-warp.org.

## Figures and Tables

**Table 1:** Validation of the method: exclusion test

Completeness of the initial model (%)	95	90	85	80	75	70	65	60	55	50
Percentage of the model excluded (%)	5	10	15	20	25	30	35	40	45	50
Residues retrieved	17	34	52	69	86	103	121	107	122	126
R.m.s.d. of the retrieved structure to C^α^ atoms of the reference model (Å)	0.08	0.09	0.14	0.21	0.29	0.30	0.32	0.31	0.37	0.45
Completeness of the retrieved structure (%)	100	100	100	100	100	100	100	91	90	87

## References

[bb1] Adams, P. D. *et al.* (2010). *Acta Cryst.* D**66**, 213–221.

[bb2] Berman, H. M., Westbrook, J., Feng, Z., Gilliland, G., Bhat, T. N., Weissig, H., Shindyalov, I. N. & Bourne, P. E. (2000). *Nucleic Acids Res.* **28**, 235–242. 10.1093/nar/28.1.235 PMC102472 10592235

[bb3] Bricogne, G. (1974). *Acta Cryst.* A**30**, 395–405.

[bb5] Cowtan, K. (2006). *Acta Cryst.* D**62**, 1002–1011. 10.1107/S0907444906022116 16929101

[bb6] Holton, J. M. (2005). *Annual Meeting of the American Crystallo­graphic Association* Abstract W0308.

[bb7] Kearsley, S. K. (1989). *Acta Cryst.* A**45**, 208–210.

[bb8] Kleywegt, G. J. (1996). *Acta Cryst.* D**52**, 842–857. 10.1107/S0907444995016477 15299650

[bb9] Kleywegt, G. J. & Read, R. J. (1997). *Structure*, **5**, 1557–1569. 10.1016/s0969-2126(97)00305-5 9438862

[bb10] Kumar, P., Singh, M. & Karthikeyan, S. (2011). *Acta Cryst.* D**67**, 131–139. 10.1107/S0907444910053370 21245535

[bb11] Langer, G., Cohen, S. X., Lamzin, V. S. & Perrakis, A. (2008). *Nature Protoc.* **3**, 1171–1179. 10.1038/nprot.2008.91 PMC2582149 18600222

[bb12] Ling, H., Boodhoo, A., Hazes, B., Cummings, M. D., Armstrong, G. D., Brunton, J. L. & Read, R. J. (1998). *Biochemistry*, **37**, 1777–1778. 10.1021/bi971806n 9485303

[bb13] Mackay, A. L. (1984). *Acta Cryst.* A**40**, 165–166.

[bb14] Morris, R. J., Perrakis, A. & Lamzin, V. S. (2003). *Methods Enzymol.* **374**, 229–244. 10.1016/S0076-6879(03)74011-7 14696376

[bb15] Morris, R. J., Perrakis, A. & Lamzin, V. S. (2007). *Macromolecular Crystallograpy: Conventional and High-throughput Methods*, edited by M. R. Sanderson & J. V. Skelly, pp. 155–172. Oxford University Press.

[bb16] Murshudov, G. N. (2011). *Acta Cryst.* A**67**, C134.

[bb17] Murshudov, G. N., Skubák, P., Lebedev, A. A., Pannu, N. S., Steiner, R. A., Nicholls, R. A., Winn, M. D., Long, F. & Vagin, A. A. (2011). *Acta Cryst.* D**67**, 355–367. 10.1107/S0907444911001314 PMC3069751 21460454

[bb18] Poon, B. K., Grosse-Kunstleve, R. W., Zwart, P. H. & Sauter, N. K. (2010). *Acta Cryst.* D**66**, 503–513. 10.1107/S0907444910001502 PMC2865365 20445225

[bb19] Rose, P. W., Beran, B., Bi, C., Bluhm, W. F., Dimitropoulos, D., Goodsell, D. S., Prlic, A., Quesada, M., Quinn, G. B., Westbrook, J. D., Young, J., Yukich, B., Zardecki, C., Berman, H. M. & Bourne, P. E. (2011). *Nucleic Acids Res.* **39**, D392–D401. 10.1093/nar/gkq1021 PMC3013649 21036868

[bb20] Rossmann, M. G. (1972). *The Molecular Replacement Method.* New York: Gordon & Breach.

[bb21] Rossmann, M. G. (2001). *Acta Cryst.* D**57**, 1360–1366. 10.1107/s0907444901009386 11567146

[bb22] Stroud, R. M., Choe, S., Holton, J., Kaback, H. R., Kwiatkowski, W., Minor, D. L., Riek, R., Sali, A., Stahlberg, H. & Harries, W. (2009). *J. Struct. Funct. Genomics*, **10**, 193–208. 10.1007/s10969-008-9058-3 PMC2705707 19148774

[bb23] Terwilliger, T. C. (2002). *Acta Cryst.* D**58**, 2082–2086. 10.1107/S0907444902016360 PMC2745884 12454468

[bb24] Terwilliger, T. C., Grosse-Kunstleve, R. W., Afonine, P. V., Moriarty, N. W., Zwart, P. H., Hung, L.-W., Read, R. J. & Adams, P. D. (2008). *Acta Cryst.* D**64**, 61–69. 10.1107/S090744490705024X PMC2394820 18094468

[bb25] Tête-Favier, F., Rondeau, J.-M., Podjarny, A. & Moras, D. (1993). *Acta Cryst.* D**49**, 246–256. 10.1107/S090744499200773X 15299530

[bb4] Winn, M. D. *et al.* (2011). *Acta Cryst.* D**67**, 235–242.

[bb27] Zhang, Y. & Skolnick, J. (2005). *Nucleic Acids Res.* **33**, 2302–2309. 10.1093/nar/gki524 PMC1084323 15849316

